# Neutrophil extracellular traps in ischemic stroke: mechanisms, clinical implications, and therapeutic potential

**DOI:** 10.3389/fneur.2025.1641985

**Published:** 2025-09-12

**Authors:** Wenbo He, Zuoli Wu, Ying Liu, Ziming Ye

**Affiliations:** ^1^Department of Neurology, The First Affiliated Hospital of Guangxi Medical University, Nanning, China; ^2^Department of Neurology, Jiangbing Hospital, Nanning, China; ^3^Department of Rehabilitation Medicine, The Second Affiliated Hospital of Guangxi Medical University, Nanning, China

**Keywords:** neutrophil extracellular traps, ischemic stroke, neuroinflammation, biomarkers, therapeutic targets

## Abstract

Ischemic stroke remains a leading cause of mortality and disability, with many patients failing to benefit from reperfusion therapies due to lysis-resistant thrombus formation and severe neuroinflammation. This highlights an urgent need to target the fundamental mechanisms linking these two processes. Neutrophil extracellular traps (NETs)—web-like structures of DNA and cytotoxic proteins—have emerged as a critical mediator of stroke pathology. While essential for host defense, their dysregulated formation in the cerebral microvasculature drives a vicious cycle of tissue injury. This review synthesizes evidence demonstrating that NETs are not mere bystanders but active drivers of stroke pathology. We dissect the core mechanisms by which they mediate three primary detrimental effects: (1) promoting stable, lysis-resistant thrombi, which directly contributes to poor clinical outcomes; (2) compromising blood–brain barrier integrity; and (3) amplifying the neuroinflammatory cascade. Furthermore, we evaluate the clinical utility of NETs as powerful biomarkers for diagnosis and prognosis, and we critically analyze emerging therapeutic strategies aimed at dismantling them. While targeting NETs with agents like DNase I or PAD4 inhibitors holds immense promise, we argue that significant translational challenges—such as ensuring therapeutic specificity and defining the optimal treatment window—must be overcome. In conclusion, targeting the thrombo-inflammatory functions of NETs represents a paradigm shift from a purely fibrin-centric view of stroke, opening new avenues for developing more effective therapies.

## 1 Introduction

Ischemic stroke, a leading cause of adult disability and mortality worldwide, is characterized by complex pathological mechanisms. In recent years, neutrophil extracellular traps (NETs) have garnered significant attention in ischemic stroke research. NETs are web-like structures composed of DNA, proteins, and enzymes released by neutrophils upon activation. These structures not only play critical roles in infection and inflammation but also contribute to the pathogenesis of diverse diseases (1–3). Accumulating evidence demonstrates elevated NET levels in brain tissues and thrombi of ischemic stroke patients, suggesting their involvement in disease progression (2, 4, 5).

While the precise mechanisms underlying NET formation and their specific roles in ischemic stroke remain incompletely understood, preclinical studies using murine models and clinical sample analyses have revealed spatiotemporal characteristics and potential pathways (6, 7). Furthermore, research on NETs in other diseases, such as cardiovascular disorders, infections, and autoimmune conditions, provides valuable insights into their multifunctional roles in health and disease (8–10), underscoring their significance in pathological processes. Emerging studies on NETs as biomarkers and therapeutic targets highlight the potential of modulating NET formation or degradation as a novel therapeutic strategy for ischemic stroke (11–13).

This review systematically explores the mechanisms, clinical implications, and advances in NET-based biomarkers and therapies. By integrating foundational research with cutting-edge clinical data, we aim to elucidate the diagnostic and therapeutic potential of NETs in ischemic stroke management.

## 2 Overview of neutrophil extracellular traps (NETs)

### 2.1 Definition and formation mechanisms

Neutrophil extracellular traps (NETs) are web-like chromatin structures released by activated neutrophils, primarily composed of decondensed DNA, histones, and granular enzymes. These structures exhibit antimicrobial properties by trapping and killing pathogens. In ischemic stroke, NET formation mechanisms and their pathological roles have garnered significant attention. A 2015 study by Grabcanovic Musija et al. (14) investigating neutrophilic inflammation and NET formation in chronic obstructive pulmonary disease (COPD) revealed conserved NET-driven pathways across inflammatory lung diseases. This finding suggests that NET biology may share common regulatory mechanisms in diverse inflammatory contexts, offering insights into their potential roles in ischemic stroke.

Furthermore, Chen et al. (13) research demonstrated that excessive NET formation exacerbates ischemic stroke outcomes, while traditional Chinese medicine (TCM) interventions targeting NET suppression could mitigate neurological damage. This dual perspective highlights the therapeutic potential of modulating NET dynamics and underscores the need for mechanistic exploration.

### 2.2 NETs in health and disease: dual roles and clinical implications

NETs play paradoxical roles in health and disease. Rystwej et al. (15) studies established NETs as central mediators in infection control, autoimmune dysregulation, cancer progression, and even reproductive physiology, revolutionizing understanding of neutrophil biology.

However, dysregulated NET activity is implicated in pathological conditions. For instance, van Dam et al. (16) work identified NETs as key contributors to autoimmune kidney diseases, including acute glomerulonephritis and systemic lupus erythematosus (SLE). Similarly, Arazna et al. (8) research linked ROS-dependent NETosis to autoimmune pathogenesis. Clinically, NET detection methods have advanced significantly. Aslanova et al. (17) study revealed NETs’ dual role in gynecological malignancies—combating infections while paradoxically driving inflammation and cancer progression. Epshtein et al. (18) work further demonstrated NETs’ utility as high-iodine X-ray contrast agents for endovascular thrombectomy (EVT) imaging, enhancing thrombus visualization.

Despite their benefits, NET dysregulation poses risks. Retter et al. (19) review emphasized that both excessive and insufficient NET activity disrupt immune homeostasis, contributing to sepsis and organ damage.

In summary, NETs represent a double-edged sword: while essential for host defense, their uncontrolled release drives pathological inflammation and thrombosis. Balancing NET activity through targeted therapies—such as peptidyl arginine deiminase 4 (PAD4) inhibitors or deoxyribonuclease I (DNase I)—holds promise for treating ischemic stroke and other NET-related disorders.

## 3 NETs in ischemic stroke

### 3.1 NET levels in ischemic stroke patients

Neutrophil extracellular traps (NETs) are significantly elevated in ischemic stroke patients. Vallés et al. (4) demonstrated that acute ischemic stroke (AIS) patients exhibit markedly increased NET levels, particularly in those over 65 years of age with atrial fibrillation or a history of cardioembolic sources. De Wilde et al. (6) further revealed that NET formation peaked within 24 h post-stroke and gradually declined by 48 h in murine ischemic stroke models.

Laridan’s (2) study investigated NET dynamics within ischemic stroke thrombi, aiming to optimize thrombolytic therapy efficacy. Lapostolle et al. (5) research identified a strong correlation between thrombus-bound NETs and unsuccessful recanalization or prolonged procedural time in mechanical thrombectomy. These findings underscore NETs’ pivotal role in ischemic stroke pathophysiology. Regarding neutrophil-to-lymphocyte ratio (NLR), Liu et al. (20) cohort study demonstrated that elevated NLR predicted poorer 90-day functional independence in young adults post-AIS or transient ischemic attack (TIA). Song et al. (21) meta-analysis systematically evaluated baseline NLR’s prognostic utility across acute stroke subtypes, while Quan et al. (22) highlighted NLR’s limitations as a standalone biomarker due to confounding variables. Yu et al. (23) work further validated NLR’s association with cardiovascular mortality and early clinical outcomes in AIS patients.

Additional NET-associated biomarkers have emerged. Lozano et al. (24) observed heightened NET formation in immune thrombocytopenia (ITP) patients with platelet and neutrophil hyperactivation. Xiao et al. (25) identified neutrophil gelatinase-associated lipocalin (NGAL) as a novel biomarker for acute kidney injury (AKI) in AIS, correlating with elevated NGAL levels in AKI cohorts. Demyanets et al. (9) linked NET biomarkers H3Cit and cfDNA to major adverse cardiovascular events (MACE) post-percutaneous coronary intervention (PCI).

In summary, robust evidence confirms elevated NET levels in ischemic stroke patients, tightly linked to adverse clinical outcomes. These insights not only elucidate NETs’ pathological contributions but also unveil potential therapeutic targets for mitigating ischemic injury.

### 3.2 NETs and clinical prognosis

Investigations into the relationship between neutrophil extracellular traps (NETs) and clinical outcomes revealed critical insights. Schechter et al.’s (26) study demonstrated a correlation between NET levels in human plasma and disease severity and antibiotic treatment responses in active tuberculosis (TB). While this research primarily focused on TB, its findings suggested that NETs play significant roles in infectious diseases, providing a foundation for future investigations into NETs’ prognostic roles in other conditions, including ischemic stroke.

### 3.3 The influence of systemic comorbidities on NETs dynamics in ischemic stroke

Ischemic stroke patients rarely present without systemic comorbidities, which may function not merely as passive risk factors but as active modulators of the innate immune response. A compelling hypothesis is that chronic conditions such as diabetes and obesity create a systemic environment that “primes” circulating neutrophils. This priming renders them hyper-responsive, poised for an exaggerated NETotic response upon the acute challenge of cerebral ischemia (27, 28).

This priming phenomenon is particularly evident in the context of metabolic disorders. Emerging evidence delineates a gut-centric mechanism where high-fat diets promote dysbiosis, which in turn compromises intestinal barrier integrity. This leads to the systemic spillover of bacterial components like lipopolysaccharide, fueling a chronic, low-grade state of “meta-inflammation” (29). Concurrently, at the cellular level, neutrophils from obese models undergo significant metabolic reprogramming, exhibiting an altered dependence on glycolysis and fatty acid oxidation (30). This two-pronged mechanism—a pro-inflammatory systemic milieu coupled with intrinsic cellular alterations—translates into clinically observable phenomena. Indeed, studies consistently detect elevated circulating markers of NETosis in patients with type 2 diabetes and obesity, confirming a state of heightened neutrophil activation (29, 31).

Beyond metabolic syndrome, this principle of neutrophil priming by chronic disease likely extends to other systemic inflammatory conditions. In systemic sclerosis, for instance, neutrophils exhibit a heightened propensity for NET formation, particularly in patients with severe vascular complications, suggesting that chronic endothelial damage and autoimmune activation may similarly prepare neutrophils for a rapid and potent NETotic response (32).

Therefore, in a patient with underlying comorbidities, circulating neutrophils can be envisioned as being “pre-activated.” When an ischemic stroke occurs, this acute, potent stimulus acts on a cell population already poised for action. The result is a fulminant and disproportionate release of NETs within the cerebral microvasculature. This excess of NETs can exacerbate thrombus stability, promote microvascular occlusion, and amplify the cascade of neuroinflammation, potentially contributing to poorer functional outcomes.

### 3.4 Clinical implication: NETs drive thrombolysis resistance

A critical clinical implication of NETs in ischemic stroke is their role in mediating resistance to thrombolytic therapy. The dense scaffold of extracellular DNA, histones, and enzymes that constitutes NETs creates a physically robust thrombus that is poorly susceptible to degradation by standard fibrin-centric lytic agents like recombinant tissue plasminogen activator (r-tPA) (33, 34). This mechanistic understanding is strongly supported by direct clinical observations. Multiple studies have now demonstrated that a high abundance of NETs within retrieved thrombi is significantly associated with poor interventional outcomes, including unsuccessful recanalization (often defined as mTICI scores <2b) and prolonged procedure times during mechanical thrombectomy (5).

Furthermore, the age of the thrombus appears to be a pivotal factor. The work by Mengozzi et al. (33) revealed that older, more organized thrombi contain a higher burden of NETs, and this thrombus age was the sole independent predictor of NET content, which in turn correlated with impaired clinical outcomes. This suggests that as thrombi mature, they become increasingly fortified by NETs, solidifying their resistance to lysis. These findings collectively underscore that conventional thrombolysis is often insufficient for NET-rich clots and have catalyzed interest in adjunct therapies. The use of DNase I, which directly degrades the DNA backbone of NETs, is emerging as a promising strategy to dismantle the thrombus scaffold, potentially restoring its susceptibility to tPA and improving overall reperfusion success (7, 33). Therefore, targeting NETs represents a paradigm-shifting approach to overcoming thrombolysis resistance in a significant subset of acute ischemic stroke patients.

## 4 Mechanistic insights into NETs in ischemic stroke

Neutrophil extracellular traps (NETs) are not mere bystanders in ischemic stroke; they are active and potent drivers of a multifaceted pathological cascade. Understanding the mechanisms by which NETs exert their detrimental effects requires a hierarchical approach, tracing their journey from the initial triggers within the ischemic microenvironment to the complex intracellular machinery of NETosis, and finally to their destructive downstream consequences on the neurovascular unit. This chapter will systematically dissect this process, providing a mechanistic framework that directly corresponds to the core pathological events in stroke.

### 4.1 Upstream triggers and intracellular pathways of NETosis

The formation of NETs in ischemic stroke is a two-stage process, beginning with potent triggers from the hostile ischemic environment that subsequently activate specific intracellular enzymatic pathways within the neutrophil. The initial stimulus arises from a combination of oxidative stress, cellular debris, and intense crosstalk with other activated cells, particularly platelets. Cerebral ischemia followed by reperfusion initiates a burst of reactive oxygen species (ROS), a well-established trigger for NETosis that directly activates key enzymes like NADPH oxidase (35, 36). Concurrently, necrotic neurons and damaged endothelium release damage-associated molecular patterns (DAMPs), such as high-mobility group box 1 (HMGB1), which function as critical danger signals. HMGB1, whether from damaged tissue or activated platelets, is a powerful inducer of NET formation, linking sterile injury to a robust innate immune response (1, 37). This process is further amplified by direct physical and biochemical interactions with activated platelets, which themselves promote NET release and functionally integrate the processes of thrombosis and inflammation (1, 36).

Once these external triggers are sensed, neutrophils execute the NETosis program via distinct intracellular cascades. The classical pathway is NADPH oxidase-dependent, where an internal ROS burst leads to membrane disintegration and allows granular enzymes like neutrophil elastase and myeloperoxidase (MPO) to access and process nuclear chromatin, culminating in cell lysis and the explosive release of NETs. A second, often faster pathway relies on the nuclear enzyme PAD4. PAD4 catalyzes histone citrullination, a key modification that neutralizes histone charges and causes profound chromatin decondensation, a prerequisite for NET externalization (36). This PAD4-dependent mechanism is a critical element in stroke-associated thrombosis and represents a key therapeutic target (35). The execution of these pathways ultimately unleashes the NET scaffold into the neurovascular space, where it orchestrates subsequent pathological events.

### 4.2 Downstream pathological effects of NETs in cerebral injury

Once formed, NETs are not inert scaffolds but are biochemically active structures that orchestrate tissue damage through three primary, interconnected mechanisms: promoting thrombosis, compromising the blood–brain barrier, and amplifying neuroinflammation.

First and foremost, NETs are profoundly prothrombotic. Their web-like DNA backbone provides a physical scaffold for the aggregation of platelets and red blood cells, promoting thrombus growth and stability (36). Biochemically, NET components activate coagulation pathways and enhance thrombin generation. This interplay is further highlighted by the fact that fibrinogen, a key clotting protein, readily deposits within the NET meshwork, strengthening the thrombus. This dual physical and chemical contribution makes the resulting thrombus incredibly stable and resistant to both endogenous and therapeutic thrombolysis. Indeed, clinical evidence compellingly shows that high NET content in retrieved stroke thrombi is a strong predictor of resistance to tPA, failed recanalization, and ultimately, poorer patient outcomes (5). Supporting the therapeutic potential of targeting this nexus, preclinical studies have demonstrated that agents like batroxobin, which can both directly inhibit NET formation and degrade fibrinogen, significantly attenuate ischemic tissue damage and improve microcirculation in peripheral ischemia models (38).

Second, NETs directly attack the integrity of the blood–brain barrier (BBB). The proteases embedded within the NET structure, such as neutrophil elastase and matrix metalloproteinases, can enzymatically degrade the tight junction proteins and basement membrane that form the seal of the BBB (35). In parallel, NET-associated histones are directly cytotoxic to endothelial cells. Furthermore, emerging evidence suggests that NETs can modulate cellular functions at the genetic level, for instance, by regulating the expression of long non-coding RNAs like NEAT1, which has been shown to influence signaling pathways that could similarly impact BBB integrity in pathological contexts (39). This combined enzymatic and cytotoxic assault creates breaches in the neurovascular barrier, leading to vasogenic edema and facilitating the influx of more inflammatory cells into the delicate brain parenchyma.

**Figure 1:**
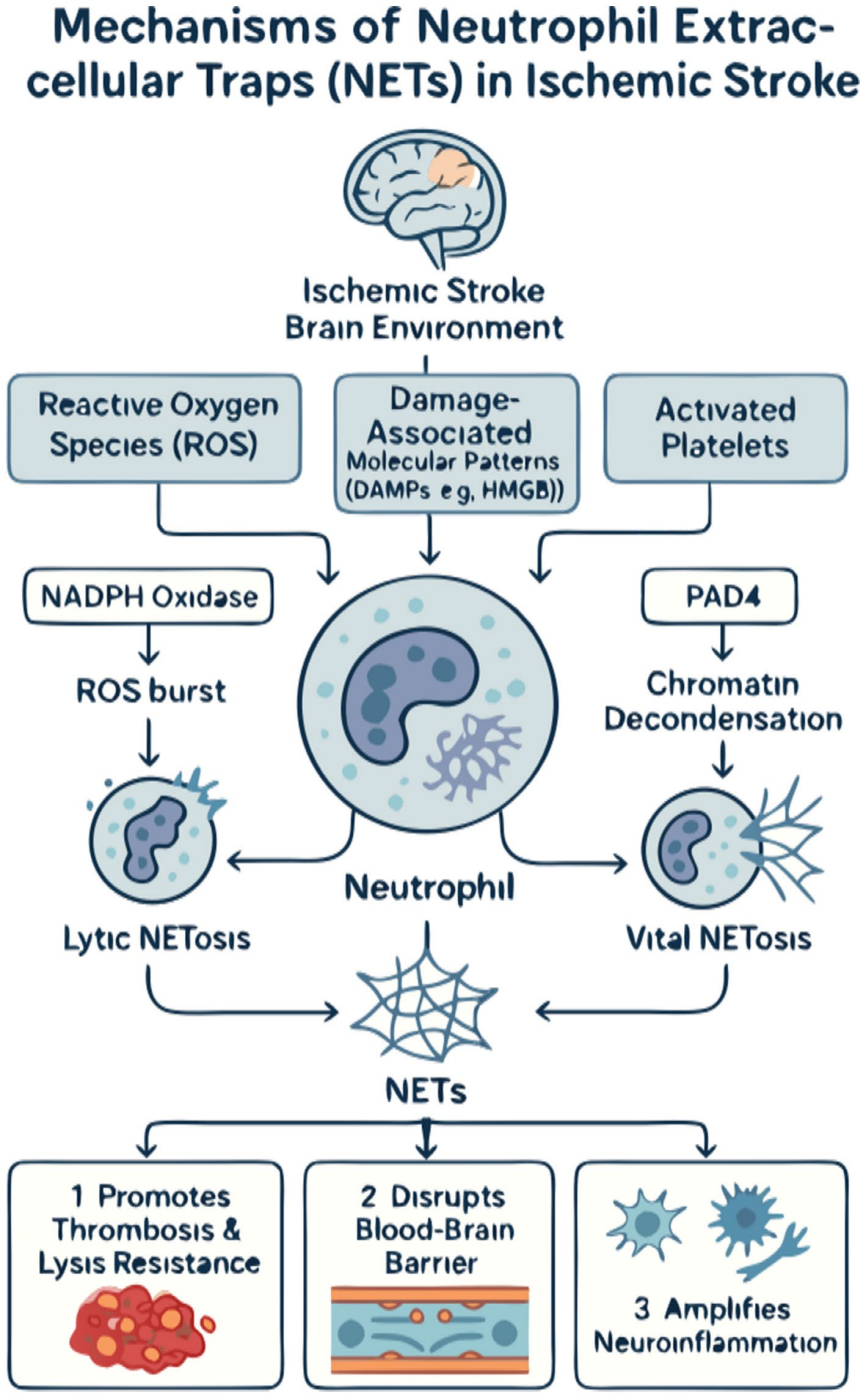
The mechanistic landscape of neutrophil extracellular traps (NETs) in ischemic stroke. **(A)** Upstream triggers: The ischemic microenvironment initiates NETosis through three primary stimuli. (1) Hypoxia/reperfusion injury leads to a burst of reactive oxygen species (ROS). (2) Tissue injury results in the release of damage-associated molecular patterns (DAMPs), such as high-mobility group box 1 (HMGB1). (3) Platelet activation promotes direct neutrophil–platelet interactions. These signals converge to activate neutrophils. **(B)** Intracellular machinery: Activated neutrophils form NETs via two main pathways. (1) The classical lytic pathway is NADPH oxidase-dependent, where ROS production leads to the release of granular enzymes (e.g., MPO AND NE) that process chromatin, culminating in cell lysis and NET release. (2) The PAD4-dependent pathway involves the nuclear enzyme peptidyl arginine deiminase 4 (PAD4), which citrullinates histones, leading to chromatin decondensation and the release of NETs, often through a non-lytic mechanism. **(C)** Downstream pathological effects: Once released, NETs exacerbate cerebral injury through three major mechanisms. (1) Pro-thrombotic effects: The DNA scaffold of NETs traps platelets and red blood cells, while its components activate coagulation, leading to larger, more stable thrombi that are resistant to thrombolysis. (2) Blood–brain barrier (BBB) disruption: NET-associated proteases (e.g., NE) degrade tight junction proteins, and histones exert direct cytotoxicity on endothelial cells, increasing BBB permeability. (3) Neuroinflammation amplification: NETs act as DAMPs to activate microglia and astrocytes, triggering a pro-inflammatory cytokine storm that recruits more neutrophils, thus perpetuating a vicious cycle of inflammation and secondary brain damage.

Finally, NETs perpetuate a vicious cycle of neuroinflammation. The DNA and histone components of NETs function as DAMPs themselves, activating resident brain immune cells like microglia and astrocytes (1). This activation triggers a secondary wave of cytokine and chemokine production, which serves to recruit even more neutrophils to the site of injury. Interestingly, the systemic inflammatory state of the host can profoundly influence this local response. For example, studies on remote organ injury have shown that a systemic insult like a burn can paradoxically suppress the recruitment of neutrophils and Subsequent NETosis in the lungs by altering chemokine levels such as CCL2 and CCL3 (40). This principle suggests that the degree of NET-driven neuroinflammation in stroke may not only be dictated by the local brain injury but also modulated by a patient’s pre-existing systemic inflammatory status. In this role, NETs act as a crucial mechanistic bridge, converting the initial acute ischemic injury into a sustained, amplified, and highly destructive state of chronic neuroinflammation that drives secondary brain damage (Figure 1).

## 5 NETs in other diseases

### 5.1 Infectious diseases

The role of neutrophil extracellular traps (NETs) in infectious diseases has garnered significant attention (41). Fonseca et al. (42) demonstrated that pathogenic *Entamoeba histolytica* induces NET formation in neutrophils, whereas non-pathogenic *E. dispar* does not. Additionally, Bystrzycka’s (43) study revealed that antibiotics such as clindamycin and amoxicillin impair NET formation, thereby compromising neutrophil antimicrobial function.

In cutaneous immunity, Stephan’s (44) research showed that NETs drive inflammatory responses in autoimmune skin diseases while contributing to host defense against pathogens. Similarly, Li’s (45) study investigating gouty arthritis identified NETs’ antibacterial properties as potential therapeutic targets. In COVID-19 patients, de Souza Andrade et al.’s (46) work demonstrated that resveratrol inhibits NET generation in severe cases, suggesting anti-inflammatory benefits. Meier’s (47) study further found that propofol suppresses NET formation in human neutrophils, potentially improving sepsis outcomes in ICU patients. Regarding chronic diseases, Kim and Jenne’s (48) research highlighted platelets’ critical role in immunity, pathogen clearance, leukocyte recruitment, and NET induction. Zucoloto’s (49) work elucidated platelet–neutrophil interactions, particularly in NET-driven coagulation. For specific infection models, Yost’s (50) study proposed nNIF (neonatal NET inhibitory factor), which reduces inflammation and improves outcomes by inhibiting NET formation. Datla et al.’s (51) development of a novel NET array device enabled single-cell NET quantification in infection and inflammation.

Mechanistically, Neubert et al.’s (52) study showed that blue and UVA light induce NET formation via ROS generation in human neutrophils. Nakayama (53) work revealed that tunicamycin induces NET-like structures in cultured myeloid cells, aiding NETosis research.

Clinically, Czerwińska et al. (54) study found elevated serum levels of NE-DNA, MPO-DNA, cit H3, and DNase I in psoriatic patients compared to healthy volunteers. Li et al. (55) research explored FPR1 (formyl peptide receptor 1), a NET-associated gene, for its prognostic and biological significance in osteosarcoma. Finally, Khan et al.’s (56) work linked cystic fibrosis-related CFTR gene defects to chronic neutrophil infiltration, airway damage, and increased mortality.

### 5.2 Cardiovascular diseases

The role of neutrophil extracellular traps (NETs) in cardiovascular diseases has garnered significant attention. Tang et al.’s (57) study investigated the roles of NETs in heart failure, pulmonary arterial hypertension, atrial fibrillation, and ischemia–reperfusion injury, revealing associations between these diseases and NETs while also proposing potential therapeutic avenues. Furthermore, Shirakawa and Sano’s (58) review highlighted that anti-inflammatory therapies improve cardiovascular disease outcomes, as neutrophils and their released NETs are implicated in the pathogenesis of these conditions. Collectively, these findings underscore NETs’ dual roles in driving pathological progression and offering therapeutic targets for cardiovascular diseases.

## 6 Diagnostic and therapeutic potential

### 6.1 NETs as biomarkers

In studies investigating neutrophil extracellular traps (NETs) as biomarkers, Baumann et al. (7) demonstrated that circulating NET markers reflect thrombotic composition, aiding in stroke diagnosis and therapeutic strategy development. Xu et al. (34) review comprehensively analyzed NET biomarkers’ roles in thrombosis and their clinical implications across diverse diseases, while exploring their therapeutic potential. Additionally, Cheng et al.’s (59) study identified human neutrophil peptides 1–3 (HNPs) as integral components of NET complexes, with dysfunction linked to lupus nephritis.

In genetic profiling, Zhang et al.’s (11) study utilized the GSE32472 dataset and machine learning to identify NET-associated genes and biomarkers, emphasizing their diagnostic significance. Abaricia et al. (60) research revealed that neutrophils form more NETs on stiffer PDMS substrates, with fibronectin coating enhancing this effect.

Cichon et al.’s (61) study demonstrated that copper ions modulate NET formation during endotoxemia, while ATP7 mutations reduce NET release in murine models. Fonseca et al.’s (62) work showed that *Entamoeba histolytica*-induced amoebiasis triggers NET formation in infected neutrophils. Moonen et al.’s (63) comparison between oral polymorphonuclear neutrophils (oPMNs) and classical PMNs (cPMNs) revealed that oPMNs maintain oral health via chemotaxis, phagocytosis, and NET formation.

Finally, Gkantzios et al. (64) study proposed neutrophil-to-HDL ratio (NHR) and monocyte-to-HDL ratio (MHR) as promising ischemic stroke prognosis biomarkers due to their anti-inflammatory effects and cost-effectiveness. Collectively, these findings underscore NETs’ broad applicability as biomarkers in stroke and other diseases.

### 6.2 Therapeutic strategies targeting NETs

Recent advances in targeting neutrophil extracellular traps (NETs) have revealed multiple promising therapeutic avenues. In 2024, Wang et al. (65) demonstrated that astrocyte-derived extracellular vesicles (ADEVs) exhibit therapeutic potential in ischemic stroke recovery, highlighting a novel strategy to modulate intercellular communication for improved stroke outcomes. Similarly, Yan et al.’s (66) study identified microglia-derived extracellular vesicles (M-EVs) as promising candidates for ischemic stroke treatment, further expanding the therapeutic landscape of extracellular vesicles in neurovascular diseases.

Luo et al.’s (67) review emphasized the transformative potential of extracellular vesicles (EVs) in drug delivery and immunotherapy, particularly when engineered to enhance targeting specificity. This concept was corroborated by Li et al.’s (68) findings, which demonstrated that EVs can traverse the BBB, offering a novel mechanism to improve neurological outcomes in ischemic stroke.

Regarding NETs formation mechanisms, Martinez et al. (69) proposed novel tetrahydroisoquinoline inhibitors in 2017 to study NETosis and develop therapies for related diseases. Building on this, Ivey (70) study revealed that chloroquine and its derivatives reduce NET formation by inhibiting PAD4 and autophagy, providing a new avenue for anti-infective therapies.

In clinical applications, Peña-Martínez et al.’s (12) research indicated that DNase-I lacks efficacy in dissolving fibrin-rich thrombi, underscoring the need for precise NET-targeted strategies. Ngo and Gollomp’s (71) study cautioned that excessive NET release may cause microvascular damage, necessitating cautious immunomodulatory approaches. Santocki and Kolaczkowska’s (72) work stressed the importance of understanding NET clearance mechanisms, as NETs not only trap pathogens but also contribute to diverse pathologies. Janssen et al.’s (73) review explored microbial evasion strategies against NETs, revealing insights into bacterial pathogenesis and inspiring novel antimicrobial therapies.

Concurrently, Zhu et al. (74) study highlighted extracellular vesicles (30–100 nm) as critical mediators of intercellular communication with therapeutic potential for stroke. Deng et al.’s (75) research combined electroacupuncture with human-induced pluripotent stem cell-derived EVs (iPSC-EVs), showing synergistic effects in modulating immune responses and the IL-33/ST2 axis to improve neurofunctional recovery.

Ortmann et al.’s (76) study in endotoxemia models demonstrated that neutrophil-derived EVs predominantly influence vascular interactions, deepening the understanding of EV-NET interplay. Huang et al.’s (77) research linked NETs to venous thromboembolism (VTE) in colorectal cancer, identifying elevated fibrinolytic activity and key protein overexpression as potential therapeutic targets. Finally, Jarzebska et al.’s (78) proposal suggested apheresis-based NET depletion as a viable strategy for age-related pathological changes, emphasizing the translational potential of NET-targeted therapies (Table 1).

**Table 1:** Summary of key therapeutic strategies targeting or modulating NETs.

Strategy category	Target/mechanism of action	Example agent/method	Key findings and nuances	References
1. Inhibiting NET formation	Inhibition of peptidylarginine deiminase 4 (PAD4), a key enzyme for chromatin decondensation	Chloroquine and derivatives	Reduces NET formation by inhibiting both PAD4 and autophagy.	Martinez et al. (69)
	Inhibition of upstream NETosis signaling pathways	Tetrahydroisoquinolines	Proposed as novel chemical probes to study NETosis and develop new therapies.	Li et al. (68)
2. Degrading existing NETs	DNA backbone of NETs	DNase-I	The classical method for NET degradation. However, studies show limited efficacy in dissolving fibrin-rich thrombi, highlighting the limitation of monotherapy.	Peña-Martínez et al.(12)
3. Physical removal of NETs	Circulating NET components	Therapeutic apheresis	Proposed as an extracorporeal strategy to clear circulating NETs, particularly for age-related pathologies.	Huang et al. (77)
4. EV-based indirect modulation	Neuroprotection and immunomodulation via intercellular communication	Astrocyte/microglia-derived extracellular vesicles (EVs)	Can cross the blood–brain barrier to modulate immune responses and improve neurofunctional recovery, representing a novel therapeutic platform.	Gkantzios et al.(64); Wang et al. (65); Luo et al.(67)

## 7 Research trends and future directions

In the study of neutrophil extracellular traps (NETs) in ischemic stroke, Xu et al. (3) bibliometric analysis in 2025 revealed emerging trends and critical research gaps. Studies indicate that China and the United States lead in this field, reflecting global prioritization of NETs’ role in ischemic stroke pathophysiology.

## 8 Related research highlights

Extensive investigations have explored NETs’ involvement in ischemic stroke. Kim et al.’s (79) study demonstrated that NETs and high-mobility group box 1 (HMGB1) drive inflammatory cascades during ischemic injury. Hirsch et al. (80) further linked extracellular vesicles (EVs) to neuroinflammation and microvascular dysfunction, suggesting their dual role in ischemic and hemorrhagic stroke progression.

Therapeutic advancements include Huang et al.’s findings that edaravone dexborneol mitigates NETs-mediated BBB disruption in acute ischemic stroke (AIS) (10). Astuti et al. (81) identified antioxidants like MonoHER as inhibitors of ROS-dependent NETosis, protecting endothelial cells from histone toxicity.

Beyond stroke, NETs’ implications span diverse pathologies. Heeringa et al. (82) linked anti-neutrophil cytoplasmic antibody (ANCA)-associated vasculitis to excessive NET release during active disease. Mauracher et al. (83) uncovered distinct neutrophil subsets (high-density vs. low-density) in lung cancer, with low-density neutrophils exhibiting heightened activation. Mechanistically, Soltani et al. (84) elucidated factor XIII-A’s role in NET-fibrin crosslinking, revealing novel therapeutic targets (84).

Stem cell-based interventions also emerged: Astuti et al.’s (81) study demonstrated that bone marrow mesenchymal stem cells (MSCs) suppress neutrophil ROS/MPO activity, promoting tissue repair (85). In pediatric oncology, Chen Cheng (2025) developed a NETRG-based prognostic model for acute lymphoblastic leukemia (ALL), identifying key survival-associated genes (86). Soongsathitanon et al. (87) further identified 35 differentially expressed proteins in diabetic neutrophils, correlating with glycemic control status.

Notably, Kernien et al. (88) discovered that *Candida albicans* biofilms consistently inhibit NET release, highlighting pathogen-immune evasion mechanisms. Collectively, these studies elucidate NETs’ multifaceted roles across diseases and provide critical insights for targeted therapeutic strategies.

## 9 Conclusion and future perspectives

This review has systematically elucidated the multifaceted roles of NETs in the pathophysiology of ischemic stroke, establishing their central position as a key pathological nexus linking innate immunity, thrombosis, and neurovascular unit injury. Substantial evidence substantiates that within the critical context of the acute phase of stroke and subsequent recanalization injury, the excessive formation of NETs not only exacerbates thrombus burden but also mediates resistance to thrombolysis, a clinical challenge highlighted by recent studies (5, 33). Concurrently, NETs drive the cascade of secondary brain injury by disrupting the BBB and amplifying neuroinflammation (1, 89). This central role solidifies NETs as one of the most promising, albeit complex, therapeutic targets in modern stroke research.

While this detrimental role of NETs in acute stroke is now well-established, our understanding is far from complete, presenting critical questions that must guide future investigation. A primary controversy is the potential “double-edged sword” nature of NETs. The literature overwhelmingly focuses on their acute, destructive functions, yet it remains plausible that NETs serve reparative roles in the subacute and chronic phases of recovery, perhaps by containing the lesion or modulating glial scar formation (19). Another fundamental question concerns NET heterogeneity. It is conceivable that NETs triggered by different stimuli, such as reactive oxygen species versus direct platelet interactions, possess distinct molecular compositions and functional capacities, leading to the existence of more pro-thrombotic or pro-inflammatory subtypes. This complexity extends to interactions with the broader neurovascular unit. For instance, a critical unanswered question is whether NET components modulate the phenotypic polarization of other key cells, such as astrocytes, which play a dynamic role in BBB integrity throughout stroke recovery (90).

Resolving these questions will require a shift toward deeper molecular investigation. A promising avenue is to link NET activity to the dysregulation of critical homeostatic signaling pathways, such as the Wnt/β-catenin cascade, which is essential for BBB maintenance and is known to be suppressed post-stroke (91). Furthermore, the field must aspire to a higher standard of molecular precision. The work by Feng et al. (92), which utilized an exogenous toxin to map the selective degradation of specific BBB proteins like ZO-1 and collagen IV, provides a compelling methodological paradigm. Future research should similarly leverage advanced proteomic techniques to identify the specific substrates of NET-associated proteases within the BBB, moving beyond phenomenology to precise molecular mechanisms.

Ultimately, translating these preclinical findings into effective clinical therapies presents significant, though not insurmountable, challenges. The first is achieving targeting specificity and safety, as systemic inhibition of NETs carries a risk of compromising innate immunity (12, 71). Second is the definition of the optimal therapeutic window, balancing acute intervention with the potential need to preserve long-term reparative functions. The third and most immediate challenge is the development of robust clinical diagnostics to rapidly quantify a patient’s NET burden, which could stratify those at highest risk for complications like thrombolysis resistance or those with comorbidities known to prime neutrophils (7, 34).

In conclusion, targeting neutrophil extracellular traps opens a new and highly promising therapeutic dimension for ischemic stroke. The scientific narrative is shifting from merely identifying their presence to understanding their central, integrative role. The path forward requires moving beyond asking whether to target NETs, to determining how to precisely modulate these complex and often paradoxical processes. By addressing the fundamental questions of their temporal dynamics and functional heterogeneity, and by overcoming the challenges of targeted delivery and clinical diagnostics, we can hope to harness the full therapeutic potential of modulating NETs to meaningfully improve outcomes for stroke patients worldwide.
